# Higher Levels of ANGPTL5 in the Circulation of Subjects With Obesity and Type 2 Diabetes Are Associated With Insulin Resistance

**DOI:** 10.3389/fendo.2019.00495

**Published:** 2019-07-24

**Authors:** Ghazi Alghanim, Mohamed G. Qaddoumi, Nouf Alhasawi, Preethi Cherian, Irina Al-Khairi, Rasheeba Nizam, Fadi Alkayal, Muath Alanbaei, Jaakko Tuomilehto, Jehad Abubaker, Mohamed Abu-Farha, Fahd Al-Mulla

**Affiliations:** ^1^Genetics and Bioinformatics Department, Dasman Diabetes Institute, Kuwait City, Kuwait; ^2^Biochemistry and Molecular Biology Department, Dasman Diabetes Institute, Kuwait City, Kuwait; ^3^Pharmacology and Therapeutics Department, Faculty of Pharmacy, Kuwait University, Kuwait City, Kuwait; ^4^Department of Medicine, Faculty of Medicine, Kuwait University, Kuwait City, Kuwait; ^5^Research Division, Dasman Diabetes Institute, Kuwait City, Kuwait

**Keywords:** obesity, insulin resistance, angiopoietin-like proteins, lipid metabolism, type 2 diabetes mellitus

## Abstract

**Objective:** The family of angiopoietin-like proteins (ANGPTLs) is composed of eight ANGPTLs members that are involved in regulating various metabolic processes and have been implicated in type 2 diabetes (T2D) and obesity. ANGPTL5 is an understudied member of this family that has been suggested to regulate triglyceride metabolism with a potential role in obesity. This study was designed to investigate the expression levels of ANGPTL5 protein in the circulation of subjects with obesity and T2D.

**Methods:** A total of 204 subjects were enrolled in this cross-sectional study, of which 95 had diagnosed T2D and 109 did not (non-T2D). Within the non-T2D group, 39 subjects were obese (BMI ≥ 30 Kg/m^2^) and 70 were not (BMI < 30 Kg/m^2^). Among subjects with T2D, 61 were obese and 34 were non-obese. Circulating ANGPTL5 plasma levels were measured by enzyme-linked immunosorbent assay (ELISA).

**Results:** In this study, we showed that ANGPTL5 levels were higher in the plasma of subjects with T2D [mean ± standard error of the mean (SEM): 5.78 ± 2.70 ng/mL] compared with individuals without T2D (mean ± SEM: 4.42 ± 2.22 ng/mL; *P* < 0.001). Obese and non-T2D subjects had significantly higher levels of ANGPTL5 (mean ± SEM: 5.115 ± 0.366 ng/mL) compared with non-obese, non-T2D subjects (mean ± SEM: 4.02 ± 0.271 ng/mL; *P* = 0.003). Similarly, among subjects with diagnosed T2D, those who were obese had higher ANGPTL5 plasma levels than non-obese subjects, although this difference did not reach statistical significance (*P* = 0.088). Correlation analyses revealed that ANGPTL5 levels positively associated with fasting plasma glucose (FPG), glycated hemoglobin (HbA1c), triglycerides (TGL), and insulin resistance as measured by HOMA-IR.

**Conclusion:** our data shows for the first time that circulating ANGPTL5 levels were higher in obese individuals and those with T2D. Further analysis will be required to better understand the interaction between ANGPTL5 and other metabolic related biomarkers to shed more light on its role in diabetes and obesity.

## Introduction

Type 2 Diabetes Mellitus (T2D) is a metabolic disorder characterized by an increase in circulating glucose levels, arising due to impaired insulin secretion and/or the resistance of peripheral tissue to insulin action (1). Factors such as a sedentary lifestyle and weight gain contribute to an increased demand for insulin secretion. Under normal conditions, the pancreatic beta cells increase the secretory output of insulin to meet this increased demand, through a mechanism termed “beta cell compensation.” However, when beta cell compensation fails–coupled with a decrease in peripheral insulin sensitivity—T2D is manifested (2). Obesity is a major risk factor for the development of T2D, with ~90% of T2D cases attributed to obesity (3). Another major risk factor associated with T2D is dyslipidemia. In ~60–70% of cases of obesity, dyslipidemia positively correlates with obesity (4). In fact, risk factors for the development of obesity-induced diabetic vascular complications are changes in triglycerides, and low-density and high-density lipoproteins (HDL). These lipid abnormalities are typically found in cases of metabolic syndrome (5).

Obesity-induced T2D leads to macrovascular complications—such as coronary artery disease, peripheral arterial disease, increased stroke risk, and impaired wound healing—and microvascular complications—such as diabetic retinopathy, neuropathy, and nephropathy–, which are major causes of morbidity and mortality in patients with T2D (6). A key mediator of these diabetic vascular complications is angiogenesis, a process by which new blood vessels are formed via the proliferation of existing endothelial cells. Excessive angiogenesis is a key characteristic of microvascular problems (7), while inadequate angiogenesis gives rise to macrovascular complications (8). This fine balance is mediated by many pro- and anti-angiogenic growth factors (9). The angiopoietin-like protein (ANGPTL) family is composed of eight proteins, named ANGPTL1 through to ANGPTL8 (10). These proteins show structural homology to angiopoietins and display an N-terminal coiled-coil domain and a fibrinogen-related domain toward the C-terminus, except for ANGPTL8, which lacks the latter domain (11). All ANGPTL are secreted glycoproteins with pro-angiogenic effects, despite not binding to Tie receptors (12). Some ANGPTL family members have been implicated in obesity, insulin resistance, and diabetes. ANGPTL3 and 4 are amongst the most well-studied members of this family, based on their role in regulating lipoprotein lipase activity (13, 14). Another member that has been shown to regulate lipoprotein lipase activity is ANGPTL8, through its interaction with ANGPTL3 (15). Recent findings suggest that ANGPTL5, along with ANGPTL7, may be involved in the growth of hematopoietic stem cells (16, 17). However, the role of ANGPTL5 in angiogenesis and lipid metabolism remains ill-defined and recent findings suggest that, in contrast to most ANGPTLs, ANGPTL5 may not regulate angiogenesis (18).

This study investigated the expression level of ANGPTL5 in the circulation of obese and non-obese subjects, with and without T2D, and its association with glycemic and lipid metabolic clinical markers.

## Research Design and Methods

### Study Population and Ethical Consent Statement

The study cohort comprised 204 subjects, including 95 subjects diagnosed with T2D and 109 subjects without T2D. Participants were stratified according to their Body Mass Index (BMI), and classified as non-obese (19.5 ≤ BMI <30 kg/m^2^) or obese (30 ≤ BMI < 40 kg/m^2^). All subjects signed a written informed consent before participating in the study which abides by the Declaration of Helsinki and was approved by the Ethical Review Board of Dasman Diabetes Institute. Subjects with prior major illness or taking any medication and/or supplement known to influence the body composition or bone mass were excluded from the study. Morbidly obese subjects (BMI ≥ 40 kg/m^2^) or subjects with Type 1 diabetes were also excluded from the study as previously reported (19–21).

### Blood Collection and Biochemical Measurements

Blood samples from all 204 study subjects were collected and plasma was prepared using vacutainer EDTA tubes. Plasma samples were aliquoted and stored at −80°C until assayed as described previously (22–24). Fasting plasma Glucose (FPG), triglycerides (TGL), total cholesterol (TC), low density lipoprotein (LDL) and HDL were measured with Siemens Dimension RXL chemistry analyzer (Diamond Diagnostics, Holliston, MA, USA). Glycated hemoglobin (HbA1c) levels were measured using the Variant^TM^ device (Bio-Rad, Hercules, CA, USA). Insulin resistance was calculated using the HOMA-IR formula: FPG (mmol/L) × fasting insulin (mU/L)/22.5.

### Plasma Levels of ANGPTL5

ANGPTL5 plasma levels were measured using ELISA kit from Wuhan EIAAB Science Co. Ltd (China). Plasma samples were thawed on ice and centrifuged at 10,000 g for 5 min at 4°C to remove any debris (22–24). The ELISA kit was validated using recombinant ANGPTL5 at a known concentration in the plasma. A plasma dilution of 1:25 showed linearity and was used in the assay. Intra-assay coefficients of variation were 7.5 to 9.2%, while the inter-assay coefficients of variation were 7.9 to 9.6%.

### Plasma Level of Obesity Biomarkers

Plasma levels of leptin, adiponectin, and plasminogen activator inhibitor (PAI) were measured by multiplexing immunobead array as outlined by the manufacturer's instructions (R&D Systems, MN, USA). The data were processed using the Bio-Plex Manager Software version 6 (Bio-Rad, CA, USA), with five-parametric curve fitting. High Sensitivity C-Reactive Protein (HsCRP) was measured by ELISA as previously reported (25, 26).

### Statistical Analysis

Comparisons between non-obese and obese subjects were made by Student's *t*-test. Comparisons between non-obese non-T2D, non-obese T2D, and obese T2D subjects were made by one-way ANOVA. Spearman's correlation coefficients were estimated to determine the associations between ANGPTL5 levels and glycaemic and metabolic biochemical variables. All data are reported as mean ± standard error of the mean (SEM). Statistical assessments were two-sided and considered to be significant when *P* < 0.05. All analyses were performed using SAS (version 9.r; SAS Institute, Cary, NC).

## Results

### Study Population Characteristics

The clinical and biochemical characteristics of the study subjects are outlined in Table 1. Subjects with T2D had significantly higher BMI, age, waist/hip ratio, TGL, FPG, HbA1c, HsCRP, and insulin levels, and significantly lower adiponectin and HDL levels (*P* < 0.05). On the other hand, total cholesterol, LDL, and leptin were not significantly different among subjects with and without T2D. Tables 2, 3 present the characteristics of obese and non-obese subjects with and without T2D. Leptin was significantly increased in obese subjects compared to non-obese subjects, regardless of T2D status. Table 4 presents the characteristics of non-obese non-T2D, obese non-T2D, and obese T2D subjects.

**Table 1:** Demographics and characteristics of the study based on their diabetes status.

	**Non-diabetic**	**T2D**	***P*-value**
	***N* = 109**	***N* = 95**	
Age (years)	42.3 ± 1.8	52.5 ± 1.0	<0.001
BMI (kg/m^2^)	28.26 ± 0.50	31.57 ± 0.43	<0.001
Waist/Hip Ratio	0.855 ± 0.01	0.96 ± 0.02	<0.001
TC (mmol/L)	5.11 ± 0.09	4.88 ± 0.14	0.184
HDL (mmol/L)	1.36 ± 0.04	1.16 ± 0.05	0.002
LDL (mmol/L)	3.21 ± 0.09	2.99 ± 0.12	0.142
TGL (mmol/L)	1.22 ± 0.09	1.67 ± 0.12	0.004
FPG (mmol/L)	5.33 ± 0.12	7.94 ± 0.29	<0.001
HbA1c (DCCT%)	5.60 ± 0.07	7.60 ± 0.18	<0.001
Insulin (U/L)	9.44 ± 066	15.14 ± 1.2	<0.001
Leptin (μg/mL)	6.67 ± 0.46	6.82 ± 0.48	0.816
Adiponectin (μg/mL)	4.99 ± 0.28	3.78 ± 0.33	0.005
HsCRP (μg/mL)	2.05 ± 0.18	3.82 ± 0.31	<0.001

*Data are presented as mean ± SEM. P-values were calculated using Student's t-test. BMI, body mass index; FPG, fasting plasma glucose; HbA1c, glycated hemoglobin; HDL, high-density lipoprotein; HsCRP, high sensitivity c-reactive protein; LDL, low-density lipoprotein; N, number of subjects; TC, Total cholesterol; TGL, triglycerides*.

**Table 2:** Demographics and characteristics of subjects without T2D.

	**Non-obese**	**Obese**	***P-*value**
	***N* = 70**	***N* = 39**	
Age (years)	40.0 ± 1.4	46.4 ± 2.1	0.01
BMI (kg/m^2^)	25.1 ± 0.4	33.9 ± 0.5	<0.001
Waist/Hip Ratio	0.83 ± 0.02	0.89 ± 0.02	0.01
TC (mmol/L)	5.07 ± 0.11	5.18 ± 0.17	0.579
HDL (mmol/L)	1.39 ± 0.05	1.31 ± 0.05	0.299
LDL (mmol/L)	3.14 ± 0.10	3.33 ± 0.15	0.31
TGL (mmol/L)	1.20 ± 0.13	1.25 ± 0.10	0.791
FPG (mmol/L)	5.22 ± 0.16	5.53 ± 0.17	0.183
HbA1c (DCCT %)	5.54 ± 0.09	5.70 ± 0.10	0.24
Insulin (U/L)	9.18 ± 0.78	10.23 ± 1.23	0.474
Leptin (μg/mL)	5.42 ± 0.47	9.12 ± 0.86	<0.001
Adiponectin (μg/mL)	5.29 ± 0.36	4.46 ± 0.43	0.15
HsCRP (μg/mL)	1.69 ± 0.20	2.76 ± 0.32	0.006

*Data are presented as mean ± SEM. P-values were calculated using Student's t-test. BMI, body mass index; FPG, fasting plasma glucose; HbA1c, glycated hemoglobin; HDL, high-density lipoprotein; HsCRP, high sensitivity c-reactive protein; LDL, low-density lipoprotein; N, number of subjects; TC, total cholesterol; TGL, triglycerides*.

**Table 3:** Demographics and characteristics of the subjects with T2D.

	**Non-obese**	**Obese**	***P*-value**
	***N* = 34**	***N* = 61**	
Age (years)	51.4 ± 1.7	53.1 ± 1.2	0.405
BMI (kg/m^2^)	26.9 ± 0.4	34.2 ± 0.3	<0.001
Waist/Hip Ratio	0.92 ± 0.02	0.98 ± 0.03	0.069
TC (mmol/L)	4.89 ± 0.29	4.88 ± 0.15	0.996
HDL (mmol/L)	1.24 ± 0.11	1.11 ± 0.04	0.292
LDL (mmol/L)	3.04 ± 0.24	2.97 ± 0.14	0.781
TGL (mmol/L)	1.56 ± 0.20	1.730 ± 0.16	0.494
FPG (mmol/L)	7.01 ± 0.33	8.44 ± 0.39	0.006
HbA1c (DCCT %)	6.59 ± 0.22	8.13 ± 0.22	<0.001
Insulin (U/L)	16.84 ± 2.26	14.11 ± 1.41	0.31
Leptin (μg/mL)	5.79 ± 0.71	7.70 ± 0.63	0.048
Adiponectin (μg/mL)	3.98 ± 0.82	3.67 ± 0.29	0.725
HsCRP (μg/mL)	3.08 ± 0.51	4.25 ± 0.39	0.073

*Data are presented as mean ± SEM. P-values were calculated using Student's t-test. BMI, body mass index; FPG, fasting plasma glucose; HbA1c, glycated hemoglobin; HDL, high-density lipoprotein; HsCRP, high sensitivity c-reactive protein; LDL, low-density lipoprotein; N, number of subjects; TC, total cholesterol; TGL, triglycerides*.

**Table 4:** Demographics and characteristics of non-obese non-T2D, obese non-T2D, and obese T2D subjects.

	**Non-T2D**		**T2D**	***P-*value**
	**Non-obese (*n*)**	**Obese (*n*)**	**Obese T2D (*n*)**	
Age (years)	39.96 ± 1.36 (70)	46.44 ± 2.05 (39)	53.10 ± 1.18 (61)	<0.001
BMI (Kg/m^2^)	25.11 ± 0.37 (70)	33.92 ± 0.47 (39)	34.18 ± 0.31 (61)	<0.0001
Waist/Hip Ratio	0.83 ± 0.02 (40)	0.89 ± 0.02 (27)	0.98 ± 0.03 (47)	0.0001
TC (mmol/L)	5.07 ± 0.11 (68)	5.18 ± 0.17 (39)	4.88 ± 0.15 (60)	0.343
HDL (mmol/L)	1.39 ± 0.05 (68)	1.31 ± 0.05 (39)	1.11 ± 0.04 (59)	0.0002
LDL (mmol/L)	3.14 ± 0.10 (68)	3.33 ± 0.15 (39)	2.97 ± 0.14 (58)	0.1836
TGL (mmol/L)	1.21 ± 0.13 (67)	1.25 ± 0.10 (39)	1.73 ± 0.16 (60)	0.0127
FPG (mmol/L)	5.22 ± 0.16 (67)	5.53 ± 0.17 (39)	8.44 ± 0.39 (61)	<0.0001
HBA1C (DCCT %)	5.54 ± 0.09 (63)	5.70 ± 0.10 (39)	8.12 ± 0.22 (61)	<0.0001
Insulin (U/L)	9.18 ± 0.78 (54)	10.23 ± 1.23 (18)	14.11 ± 1.41 (46)	<0.0001
Leptin (μg/ml)	5417.90 ± 465.83 (55)	9124.90 ± 856.74 (28)	7695.24 ± 625.56 (31)	0.0001
Adiponectin (μg/ml)	5285.66 ± 361.29 (62)	4464.58 ± 434.45 (34)	3669.38 ± 285.85 (56)	0.0031
HsCRP (μg/mL)	1687.09 ± 200.18 (61)	2764.78 ± 321.95 (31)	4248.08 ± 387.63 (56)	<0.0001

*Data are presented as mean ± SEM. P-values were calculated using one-way ANOVA. BMI, body mass index; FPG, fasting plasma glucose; HbA1c, glycated hemoglobin; HDL, high-density lipoprotein; HsCRP, high sensitivity c-reactive protein; LDL, low-density lipoprotein; n, number of subjects; TC, Total cholesterol; TGL, triglycerides*.

### Higher ANGPTL5 Plasma Levels of Obese Subjects and Subjects With T2D

**Figure 1:**
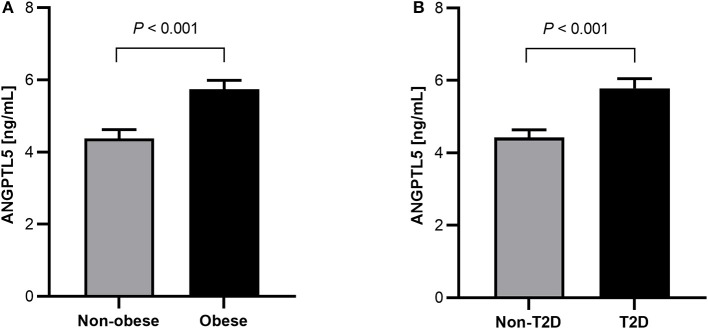
**(A)** Circulation level of ANGPTL5 in non-obese (*n* = 104) vs. obese subjects (*n* = 100) as measured by ELISA. **(B)** Circulation level of ANGPTL5 in non-diabetics (*n* = 109) vs. T2D subjects (*n* = 95) as measured by ELISA. *P* < 0.05 was considered significant as determined using student's *t*-test.

**Figure 2:**
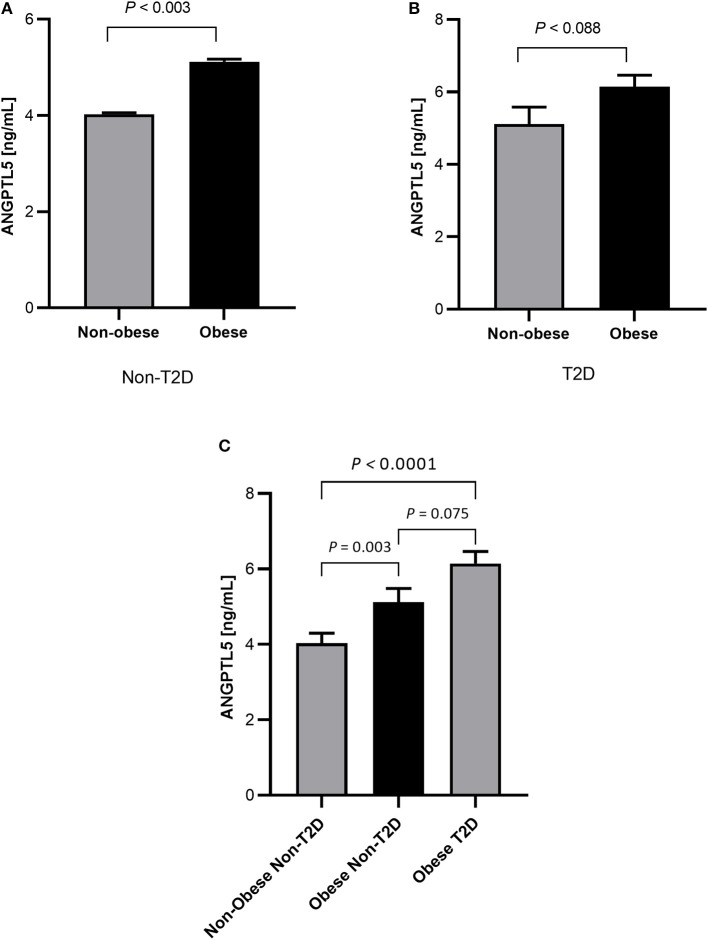
**(A)** Circulation levels of ANGPTL5 in non-obese (*n* = 70) vs. obese subjects (*n* = 39) as measured by ELISA in non-diabetic people. **(B)** Circulation levels of ANGPTL5 in non-obese (*n* = 34) vs. obese subjects (*n* = 61) as measured by ELISA in people with T2D. *P* < 0.05 was considered significant as determined using student's *t*-test. **(C)** Circulation levels of ANGPTL5 in non-obese non-T2D (*n* = 70), obese non-T2D (*n* = 39), and obese T2D (*n* = 61) as measured by ELISA. *P* < 0.05 was considered significant as determined using one-way ANOVA.

**Figure 3:**
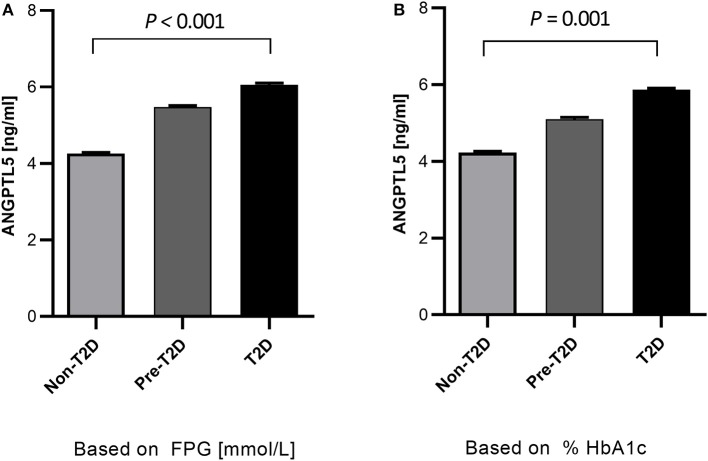
Quantitative analysis of ANGPTL5 plasma levels according to prediabetes and T2D diagnosis based on either FPG **(A)** and HbA1c **(B)**. *P* < 0.05 was considered significant as determined using student's *t*-test. Pre-diabetes level was based on ADA criteria.

Obese subjects had significantly higher levels of ANGPTL5 compared with non-obese subjects (mean ± SEM: 5.74 ± 0.25 ng/mL vs. 4.38 ± 0.24 ng/mL; *P* < 0.001; Figure 1A). Similarly, circulating levels of ANGPTL5 were higher in subjects with T2D compared with non-T2D (mean ± SEM: 5.78 ± 0.27 ng/mL vs. 4.42 ± 0.22 ng/mL; *P* < 0.001), as demonstrated in Figure 1B. In subjects without T2D, we observed that obese subjects had a significantly higher levels of ANGPTL5 compared with non-obese (mean ± SEM: 5.12 ± 0.37 vs. 4.03 ± 0.27 ng/mL; *P* = 0.003; Figure 2A). On the other hand, among subjects with T2D, those who were obese tended to have higher levels of ANGPTL5 compared with non-obese subjects (mean ± SEM: 6.15 ± 0.32 vs. 5.12 ± 0.46 ng/mL; *P* = 0.088; Figure 2B). Further analysis of the study population revealed that ANGPTL5 levels were significantly higher in obese subjects with T2D, when compared with non-obese non-T2D subjects (mean ± SEM: 4.027 ± 0.271 vs. 6.145 ± 0.317 ng/mL; *P* < 0.0001 Figure 2C). Furthermore, obese subjects with T2D had higher levels of ANGPTL5 than obese subjects without T2D, although without achieving statistical significance (mean ± SEM: 5.119 ± 0.366 vs. 6.145 ± 0.317 ng/mL, *P* = 0.075; Figure 2C). Further stratification on T2D status of patients showed that ANGPTL5 levels were higher in subjects diagnosed with pre-diabetes compared with non-T2D subjects, but lower than in subjects with T2D (Figures 3A,B).

### Correlation of ANGPTL5 Levels With Anthropometric and Clinical Markers

**Figure 4:**
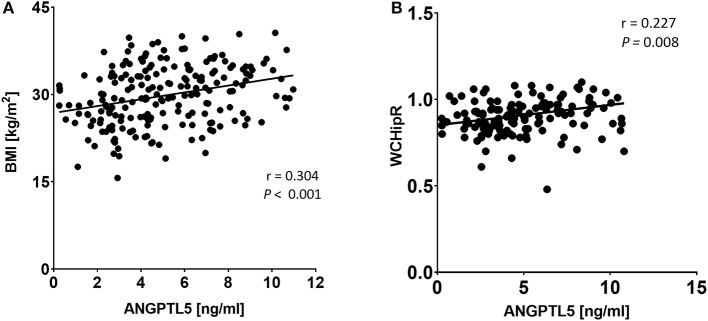
Spearman's correlation analysis of ANGPTL5 levels and BMI **(A)** and waist/hip ratio measurements **(B)**. *P* < 0.05 was considered significant as determined by Spearman's correlation.

**Figure 5:**
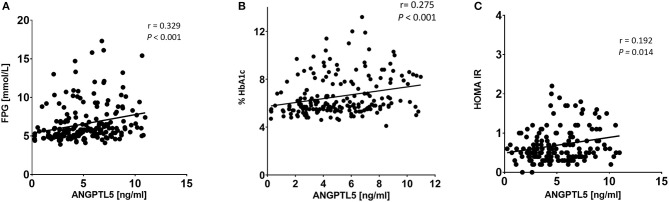
Spearman's correlation analysis of ANGPTL5 levels and blood glucose metabolites including FPG **(A)**, HbA1c **(B)**, and HOMA-IR **(C)**. *P* < 0.05 was considered significant as determined by Spearman's correlation.

**Figure 6:**
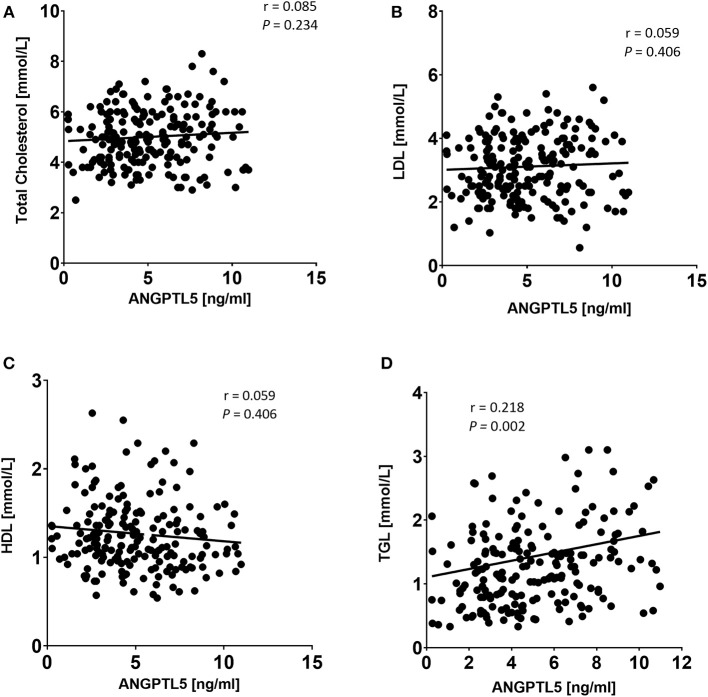
Spearman's correlation analysis of ANGPTL5 levels and TC **(A)**, LDL **(B)**, HDL **(C)**, and TGL **(D)**. *P* < 0.05 was considered significant as determined by Spearman's correlation.

**Figure 7:**
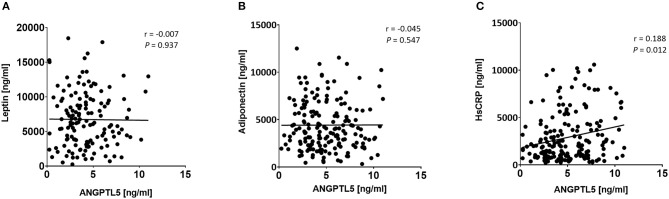
Spearman's correlation analysis of ANGPTL5 levels and obesity markers including leptin **(A)**, adiponectin **(B)**, and HsCRP **(C)**. *P* < 0.05 was considered significant as determined by Spearman's correlation.

ANGPTL5 plasma levels positively correlated with BMI (*r* = 0.304; *P* < 0.001) and waist/hip ratio (*r* = 0.227, *P* = 0.008; Figure 4). Plasma ANGPTL5 levels positively correlated with FPG (*r* = 0.329, *P* < 0.001), HbA1c (*r* = 0.275, *P* < 0.001) and insulin resistance, measured by HOMA-IR (*r* = 0.192, *P* = 0.014; Figure 5). Correlation analysis of lipid profile markers with ANGPTL5 levels showed a significant positive correlation between ANGPTL5 and plasma TGL (*r* = 0.218, *P* = 0.002), but not with total cholesterol, LDL, or HDL (Figure 6). The obesity markers leptin and adiponectin did not show any correlation with ANGPTL5 in our study population. However, higher ANGPTL5 levels correlated with higher HsCRP (*r* = 0.188; *P* = 0.012; Figure 7).

## Discussion

While ANGPTL5 has been suggested to be involved in TGL metabolism, its role in diabetes and obesity remains to be elucidated. Here, we report the results of a cross-sectional study showing the association of ANGPTL5 plasma levels with obesity and T2D. In this study, we observed higher plasma levels of ANGPTL5 in obese subjects, which positively correlated with increased BMI and waist/hip ratios. Additionally, subjects with pre-diabetes and T2D had elevated ANGPTL5 levels. ANGPTL5 plasma levels also showed positive correlation with insulin resistance as measured by HOMA-IR. There was a significant increase in TGL levels in subjects with T2D, which showed a positive correlation with ANGPTL5 levels. ANGPTL5 levels did not correlate with total cholesterol and LDL levels, or with the significant change in HDL level observed in subjects with T2D. Similarly, the significant changes observed in HsCRP in subjects with T2D and obese subjects without T2D, positively correlated with ANGPTL5 levels. The significant change in leptin in obese subjects, seen in subjects with and without T2D, did not correlate with ANGPTL5 levels. Similarly, the significant change in adiponectin levels in subjects with T2D did not correlate with ANGPTL5.

The roles of the other angiopoietin-like proteins in obesity and T2D have been studied previously, with some ANGPTLs having a marked role. Elevated levels of ANGPTL4, for example, has been shown to positively correlate with BMI, TGL, HOMA-IR, and HbA1c (27); interestingly, polymorphisms found in ANGPTL4 had been shown to positively correlate with body fat (28). In fact, genetic inactivation of ANGPTL4 was shown to reduce risk of T2D, improve insulin sensitivity and glucose homeostasis (29). Similarly, ANGPTL8 was shown to be positively associated with T2D, and correlated with many risk factors such as FPG, HbA1c, and HOMA-IR in non-T2D subjects, and thus may be a good predictor of T2D (30). In our study, subjects with T2D had significantly higher ANGPTL5 plasma levels, which positively correlated with FPG, HbA1c, and insulin resistance. While a difference was markedly evident in obese subjects without T2D when compared with non-obese subjects, in subjects with T2D we observed a non-significant elevation of ANGPTL5 in both obese and non-obese subjects. In fact, levels of ANGPTL5 found in non-obese subjects with T2D were comparable to those found in obese subjects without diagnosed T2D. The lack of significant changes in ANGPTL5 levels between obese and non-obese subjects with T2D may be due to effect of antidiabetic medication and a conscious effort to control obesity. For example, ANGPTL7 levels, previously shown to be increased in obese subjects, were shown to be significantly reduced upon exercise (19). We also observed that subjects who were at risk of developing T2D, i.e., pre-diabetes, had higher levels of ANGPTL5 than those without T2D, but lower than those with T2D. As ANGPTL5 levels also correlated positively with HbA1c, these observations, indicate that ANGPTL5 levels could be associated with poor glycemic control and increase the risk for T2D.

Previously, we have shown that obese subjects had significantly higher ANGPTL7 levels in both plasma and adipose tissue, which also correlated with increased TGL levels (19). ANGPTL2 was also found to be associated with obesity and insulin resistance, and to positively correlate with TGL levels (31). Conversely, ANGPTL6 had been shown to negatively correlate with obesity and insulin resistance, alongside a marked increase in energy expenditure (32), in a manner independent of lipoprotein lipase and lipid metabolism regulation (33). Although observed changes in LDL, HDL, and total cholesterol did not correlate with ANGPTL5, higher TGL levels significantly correlated with ANGPTL5 levels in subjects diagnosed with T2D. This positive correlation with TGL levels was previously observed with ANGPTL3,−4, and−7. Genetic work have illustrated that loss-of-function mutations in ANGPTL3,−4, and−5, but not ANGPTL6, correlates with lower TGL levels (34). As both ANGPTL3 and ANGPTL4 are important in lipid metabolism through their interaction with lipoprotein lipase (35), our data suggest a role for ANGPTL5 in TGL metabolism in obesity.

Interestingly, there were no significant correlations between ANGPTL5 and leptin. As an anti-obesity hormone (36), leptin was observed to be increased in obesity irrespective of T2D status. Whilst we observed a positive correlation between obesity and ANGPTL5, leptin did not correlate with increasing ANGPTL5. Other ANGPTL's were shown to correlate with leptin levels; ANGPTL3 is increased in leptin-resistant or leptin-deficient mice, highlighting a negative correlation with leptin (37). ANGPTL4, on the other hand, was previously shown to differentially correlate with leptin in a tissue-specific manner. In adipose tissue, leptin suppressed ANGPTL4 expression levels, whilst in the hypothalamus, leptin increased ANGPTL4 expression levels (38). Likewise, with adiponectin's role in regulating glucose levels and triglyceride clearance (39, 40), decreased adiponectin levels have been associated with the development of T2D and obesity (41). We observed a decrease in adiponectin levels in subjects with T2D in our study population, however there was no significant correlation between adiponectin and ANGPTL5. Given this lack of correlation between ANGPTL5 levels and both leptin and adiponectin, we suggest that the role of ANGPTL5 in obesity and T2D occurs independently of leptin and adiponectin.

Finally, we investigated the correlation between ANGPTL5 and HsCRP, a marker for low-grade chronic inflammation that may affect TGL metabolism and may correlate with increased TGL levels (42). HsCRP has been shown to be increased in obesity (43) and is a marker for cardiovascular disease and metabolic syndrome (44). Here, we show that HsCRP positively correlated with ANGPTL5, an observation also seen with ANGPTL3, ANGPTL4 (45), and ANGPTL7 (45). The higher HsCRP levels observed in obese subjects in our study population were statistically significant in those without T2D, but not in subjects with T2D. Serum levels of HsCRP were previously shown to be increased in obese subjects in a pre-diabetic state, indicating a role for HsCRP as a predictor for the development of T2D (46).

Whilst the functions and roles of other members of the angiopoietin-like family had been previously elucidated, ANGPTL5 function is yet to be established. As we have shown here, our data suggest that ANGPTL5 has an important role in obesity, TGL metabolism and T2D, and may be a possible indicator of a pre-diabetes state or metabolic syndrome. To the best of our knowledge, this study is the first to investigate the relationship between ANGPTL5 with obesity and T2D. However, due to the nature of observational studies, these findings have their limitations. As a non-randomized, cross-sectional study, it is limited in its predictions and thus, is only hypothesis generating. Without longitudinal data, the causal correlation between ANGPTL5 and T2D remains undefined, and therefore, mechanistic studies are required to further our understanding of the relationship of ANGPTL5 with T2D. Further functional studies to elucidate the mechanism of action of ANGPTL5, such as establishing its role in lipoprotein lipase activity, are needed to confirm its involvement in obesity and T2D and may support its apparent importance in TGL metabolism and as an indicator of a pre-T2D and predictor of T2D.

## Data Availability

The datasets generated for this study are available on request to the corresponding author.

## Ethics Statement

All subjects signed a written informed consent before participating in the study which abides by the Declaration of Helsinki and was approved by the Ethical Review Board of Dasman Diabetes Institute.

## Author Contributions

GA, MQ, and NA: data interpretation and manuscript writing. IA-K, PC, RN, and FA: ELISA assay. JT and MA: data analysis and management. JA: study design, data interpretation, and critical revision of the manuscript. MA-F: study design, data interpretation, and directed the laboratory investigation. FA-M: clinical data collection, and critical revision of the manuscript.

### Conflict of Interest Statement

The authors declare that the research was conducted in the absence of any commercial or financial relationships that could be construed as a potential conflict of interest.
